# PINK1-Parkin-Mediated Mitophagy Protects Mitochondrial Integrity and Prevents Metabolic Stress-Induced Endothelial Injury

**DOI:** 10.1371/journal.pone.0132499

**Published:** 2015-07-10

**Authors:** Weiwei Wu, Hao Xu, Zemin Wang, Yun Mao, Liangshuai Yuan, Wei Luo, Zhaoqiang Cui, Taixing Cui, Xing Li Wang, Ying H. Shen

**Affiliations:** 1 Research Center for Cell Therapy, Key Laboratory of Cardiovascular Remodeling and Function Research, Qilu Hospital of Shandong University, Jinan 250012, P.R. China; 2 Department of Cell Biology and Anatomy, South Carolina University, Columbus, South Carolina29209, United States of America; 3 Division of Cardiothoracic Surgery, Michael E. DeBakey Department of Surgery, Baylor College of Medicine, Houston, TX, United States of America; 4 Texas Heart Institute, Houston, TX, United States of America; University of Alabama at Birmingham, UNITED STATES

## Abstract

Mitochondrial injury and dysfunction, a significant feature in metabolic syndrome, triggers endothelial cell dysfunction and cell death. Increasing evidence suggests that mitophagy, a process of autophagic turnover of damaged mitochondria, maintains mitochondrial integrity. PINK1 (phosphatase and tensin homolog (PTEN)-induced putative kinase 1) and Parkin signaling is a key pathway in mitophagy control. In this study, we examined whether this pathway could protect mitochondria under metabolic stress. We found that palmitic acid (PA) induced significant mitophagy and activated PINK1 and Parkin in endothelial cells. Knocking down PINK1 or Parkin reduced mitophagy, leading to impaired clearance of damaged mitochondria and intracellular accumulation of mitochondrial fragments. Furthermore, PINK1 and Parkin prevented PA-induced mitochondrial dysfunction, ROS production and apoptosis. Finally, we show that PINK1 and Parkin were up-regulated in vascular wall of obese mice and diabetic mice. Our study demonstrates that PINK1-Parkin pathway is activated in response to metabolic stress. Through induction of mitophagy, this pathway protects mitochondrial integrity and prevents metabolic stress-induced endothelial injury.

## Introduction

Mitochondria are important organelles with diverse functions [1,2], not only in ATP production and calcium homeostasis [3], but also in reactive oxygen species (ROS) generation [4], danger signaling [2], inflammation [5] and cell death control [6–8]. Mitochondrial damage induced by metabolic stress [8] triggers endothelial dysfunction and injury, contributing to the development of cardiovascular diseases [8–10]. Preserving healthy mitochondria is essential for maintaining endothelial homeostasis and functions. However, the regulation of mitochondrial quality control in response to metabolic stress is not completely understood.

Mitophagy, a specific process of autophagic turnover of mitochondria [11], is an important mechanism of mitochondrial quality control. Mitophagy selectively removes dysfunctional or damaged mitochondria and maintains healthy mitochondria population [11–14]. Deficiency in mitophagy triggers ROS production, inflammation and cell death [5] and is linked to neurodegeneration [11] and cardiovascular [14,15] diseases.

PINK1 (phosphatase and tensin homolog-induced putative kinase 1) and Parkin pathway is a critical pathway in controlling mitophagy [13]. In healthy mitochondria, PINK1 is imported to the inner mitochondrial membrane, where it is cleaved and degraded. When mitochondria are damaged, loss of membrane potential prevents PINK1 inner membrane translocation and subsequent degradation, leading to its accumulation on the outer membrane surface where it recruits cytosolic Parkin. Parkin, an E3 ligase, induces mitophagy by promoting mitochondrial fission/mitofission to isolate the damaged mitochondrial fragments, and by ubiquitinating mitochondrial proteins to facilitate their recognition and recruitment to the autophagosomal surface [11,16,17]. Mutations in PINK1 and Parkin are associated with mitochondrial dysfunction and neurodegenerative diseases such as Parkinson’s disease [18,19].

Given the importance of PINK1-Parkin in mitophagy and mitochondrial quality control, we asked whether this protective mechanism also exists to prevent metabolic stress-induced mitochondrial dysfunction in endothelial cells. Our findings suggest that this pathway is activated in response to metabolic stress, and plays a critical role in mitochondrial protection under this condition.

## Materials and Methods

### Animal models

Animal procedures, housing and diets were conducted in accordance with National Institutes of Health guidelines of the use and care of experimental animals and approved by the Institute Animal User and Ethical Committees at Shandong University. Male C57BL/6 mice were purchased from Vital River Laboratory Animal Technology Co. Ltd, Beijing, China, and housed under a 12:12 h light-dark cycle and given free access to food and water. For induction of obesity, four week old male C57BL/6 mice were given either high-fat diet (HFD, n = 12) or chow diet (CD, n = 6) as control for 12 weeks. For induction of type 1 diabetes, eight-week old male C57BL/6J mice received daily intraperitoneal injections of either 50 mg/kg streptozotocin (STZ, n = 8) or vehicle (n = 5) as control for five consecutive days. Aortas were collected 4 weeks later, Body weight and non-fasted blood glucose levels were measured before the injection and 10 days after the injection. Mice were considered diabetic if their blood glucose levels were > 300–400 mg/dL. Mice were anesthetized with pentobarbital sodium (50 mg/kg body weight, i.p.) and euthanized by exsanguination. Blood vessels were fixed with 10% formalin at 100 mmHg for 10 min and then washed with by 0.9% NaCl for 5 min. The Aortas were harvested and further fixed with 4% phosphate-buffered formaldehyde at 4°C for 24 h, and then embedded in Frozen Section Compound (FSC 22 Clear, Leica Biosystems, Richmond, IL, USA) for sectioning.

### Cell culture and transfection

Primary human aortic endothelial cells (HAECs) (Sciencell, Carlsbad, CA) were cultured in endothelial culture medium (ECM) (Sciencell, Carlsbad, CA) containing 5% fetal bovine serum, endothelial cell growth supplements and penicillin/streptomycin. Silencing PINK1 and Parkin expression was achieved by using specific siRNAs (Genepharma, Shanghai, China). Overexpressing PINK1 and Parkin was achieved by using plasmids pCMVTNT PINK1 C-myc [20] and pMXs-IP HA-Parkin [21] (Addgene, Cambridge, MA). Transfection of HAECs was performed using LipofectAMINETM 2000 (Invitrogen, Grand Island, NY) according to the manufacturer’s instruction.

### Palmitic acid preparation and treatment

Saturated palmitic acid (PA) was used in this study. PA preparation and treatment were performed as described previously [22].

### Western blot analysis

Western blot analysis was performed as described before [22]. Primary antibodies used were anti-PINK1 (Cell Signaling, Boston, MA), anti-Parkin (Cell Signaling, Boston, MA) and anti-GAPDH (Santa Cruze, CA).

### Immunostaining and confocal imaging

Immunostaining of treated cells or aortic sections was performed as described previously [23]. The stained cells were observed using a Nikon eclipse Ti and UltraVIEW VOX confocal microscope (Nikon Instruments Inc. Melville, NY). The images were obtained under same exposure condition. Images were analyzed by using software Volocity (PerkinElmer Inc. Waltham MA). Primary antibodies used were anti-PINK1 (Abcam, Cambridge, MA), anti-Parkin (Abcam, Cambridge, MA), anti-LC3 (Cell Signaling, Boston, MA), anti-Lamp1 (abcam, Cambridge, MA).

### Detection of mitochondria

Mitochondria were detected by using MitoTracker Deep Red staining kit (Life Technologies, Carlsbad, CA). Treated cells were incubated with 50 nM MitoTracker Deep Red diluted in serum free culture medium at 37°C for 30 min, followed either by immunostaining or by continue incubation in culture medium for live cell detection. The cells were observed immediately by a Nikon eclipse Ti and UltraVIEW VOX confocal microscope (Nikon Instruments Inc. Melville, NY).

### Detection of mitophagy

Mitophagy was determined the co-localization of mitochondria with autophagosome or with lysosomome, following the standard guidelines [24]. The cells were stained with mitochondria marker (Mito), autophagosome marker microtubule-associated protein 1 light chain 3 (LC3), and lysosome marker lysosomal-associated membrane protein 1 (Lamp1). Images were captured at a high magnification (100×10) by an UltraVIEW VOX confocal microscope (Nikon Instruments Inc. Melville, NY). At least 25–30 cells per condition were captured. The number of mitochondria co-localized with LC3 or Lamp1 were quantified.

### Transmission electronic microscope (TEM)

Treated cells were incubated with 5nM bafilomycin A1 for 6h prior to harvest. The cells were then fixed with 3% glutaraldehyde at 4°C overnight and then treated with 1% osmium tetroxide at room temperature for 2h. The cells were dehydrated, embedded in resin and sectioned to a 70-nm thickness. The cells were then stained with uranylacetate and lead citrate. The ultrathin sectioned cells were observed using TEM (JEOL-1200EX) recording by MORADA-G2. The number of mitophagosomes per cell were quantified. At least 10–15 cells per condition were imaged.

### Detection of mitochondrial membrane potential

Mitochondrial membrane potential was detected by using tetramethylrhodamine methyl ester (TMRM), a cell-permeant, positively-charged, red-orange fluorescent dye that is readily sequestered by active mitochondria due to their relative negative charge. Depolarized or inactive mitochondria have decreased membrane potential and fail to sequester TMRM. In brief, cells were treated with PA or carbonylcyanide-3-chlorophenylhydrazone (CCCP, Sigma, St.Louis, Missouri). The treated cells were incubated with 20 nM TMRM (Life Technologies, Carlsbad, CA) and 50 nM MitoTracker deep red (Life Technologies, Carlsbad, CA) at 37°C for 20 min allowing dye equilibration across the plasma and inner mitochondrial membranes. The cells were washed and then observed under confocal microscope. The ratio of the TMRM fluorescence and MitoTracker was used as an indicator of mitochondrial membrane potential. All the data were presented as the average of three independent experiments.

### Detection of ROS

Intracellular ROS production was measured by using dichloro-dihydro-fluorescein diacetate (DCFH-DA) reactive oxygen species assay kit (Beyotime, Nanjing, China). The treated cells were incubated with 10 μM DCFH-DA in serum-free medium at 37°C for 30 min in the darkness. The cells were washed and then observed under confocal microscope.

Mitochondrial ROS (MitoROS) production was measured by co-staining of DCFH-DA and MitoTracker. The treated cells were incubated with 10 μM DCFH-DA and 50 nM MitoTracker in serum free culture medium at 37°C for 30 min in the darkness. The cells were washed and then observed under confocal microscope. The images were captured. Co-localization of DCFH-DA signal with MitoTracker were analyzed and quantified by using software Volocity (PerkinElmer Inc. Waltham MA, USA).

### Detection of intracellular ATP

Intracellular ATP levels were determined using an ATP Bioluminescence Assay kit (Beyotime, Nanjing, China) according to manufacturer’s protocol. ATP content was expressed as nmol/mg protein, and the data were presented as the average of three independent experiments.

### TUNEL

Apoptosis Assays Kit (Roche, Jinan, China) for TUNEL staining was used to determine cell apoptosis.

### Statistical analysis

All data were presented as means ± SD resulting from at least three independent experiments. The difference between the groups were analyzed by Student’s t test or ANOVA, as appropriate. P < 0.05 was considered statistically significant.

## Results

### Autophagy and mitophagy were increased in PA-treated cells

**Fig 1:**
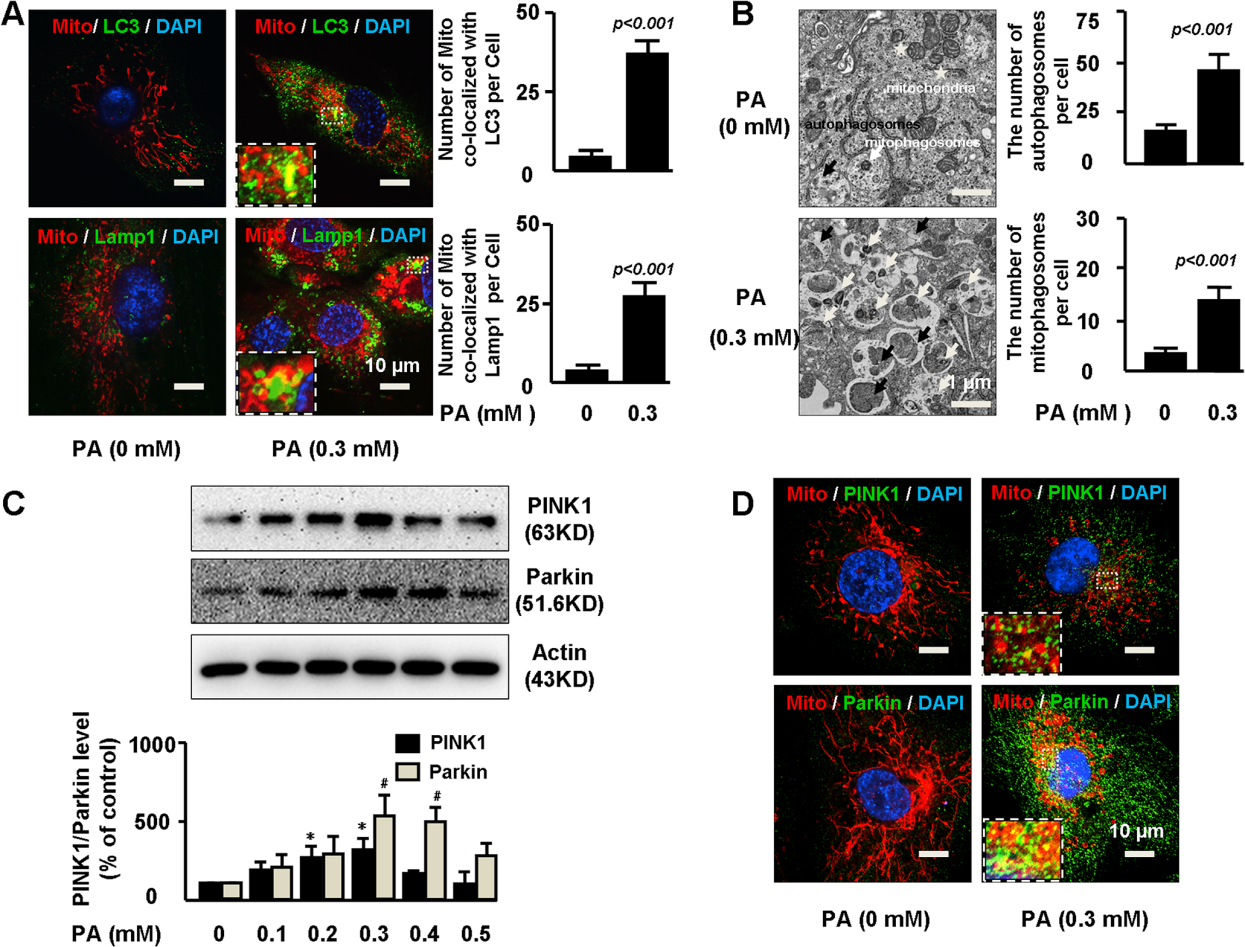
Autophagy and mitophagy were increased in PA-treated cells. **A.** Primary human aortic endothelial cells (HAECs) were treated 0 or 0.3mM palmitic acid (PA) for 24 hours. Treated cells were stained with mitochondria marker Mito, autophagy marker LC3, and lysosome marker Lamp1. The numbers of mitochondria co-localized with LC3 or with Lamp1 per cell were quantified. Representative immunostaining images and quantification of 3 independent experiments demonstrated increased co-localization of mitochondria with LC3 and Lamp1 in PA treated cells. At least 25–30 cells per experiment were analyzed. **B.** HAECs were treated with 0 or 0.3mM PA for 24h and 5nM bafilomycin A1 during the last 6h prior to fixation. Representative electron microscopy photomicrographs showed the formation of autophagosomes containing heterogeneous cytoplasmic materials (black arrows) and mitophagosomes containing mitochondria fragments (white arrows). Asterisk indicated normal mitochondria. At least 10–15 cells per condition were imaged. Quantification showed the number of autophagosome and mitophagosome was increased by PA treatment. **C.** HAECs were treated with 0 to 0.5mM PA for 24h. Representative images of western blot and quantification from 3 independent experiments showed dual effects of PA on PINK1 and Parkin expression. */#P< 0.001 vs. PA (0mM). **D.** HAECs were treated with 0 or 0.3mM PA for 24h. Representative immunostaining images of 3 independent experiments demonstrated co-localization of PINK1 and Parkin with mitochondria in perinuclear area in PA treated cells.

To determine the role of the PINK1-Parkin pathway in mitochondrial integrity under metabolic stress condition, we first examined the effect of metabolic stress on mitophagy and PINK1-Parkin activation. Human aortic endothelial cells (HAECs) were treated with palmitic acid (PA)—a main type of free fatty acid that is elevated in obese subjects with metabolic syndrome. Immunofluorescent staining showed that PA increased the level of autophagosome marker LC3 (Fig 1A), indicating the activation of autophagy in general. PA also stimulated the co-localization of mitochondria with autophagosome marker LC3 and lysosome marker Lamp1 (Fig 1A), suggesting the formation of mitophagosomes and mitochondrial autolysosomes. Consistently, electron microscopy analysis of the PA treated cells showed many autophagic vacuoles with engulfed mitochondria, confirming mitophagosome formation (Fig 1B). Interestingly, PA exhibited dual effects on PINK1-Parkin activation in human aortic endothelial cells. PA increased PINK1 and Parkin at lower dose of 0.3 mM (Fig 1C). However, PA decreased PINK1 and Parkin at higher concentration of 0.5 mM, suggesting a biphasic effects of PA on PINK1 and Parkin regulation. To focus on the effects of PINK1-Parkin on mitochondrial integrity, in this study, we used 0.3 mM PA to stimulate PINK1-Parkin expression. Immunostaining showed that PA not only increased PINK1 and Parkin levels, but also promoted PINK1 and Parkin localization to mitochondria in peri-nuclear area (Fig 1D).

### PINK1 and Parkin were involved in mitophagy in PA-treated cells

**Fig 2:**
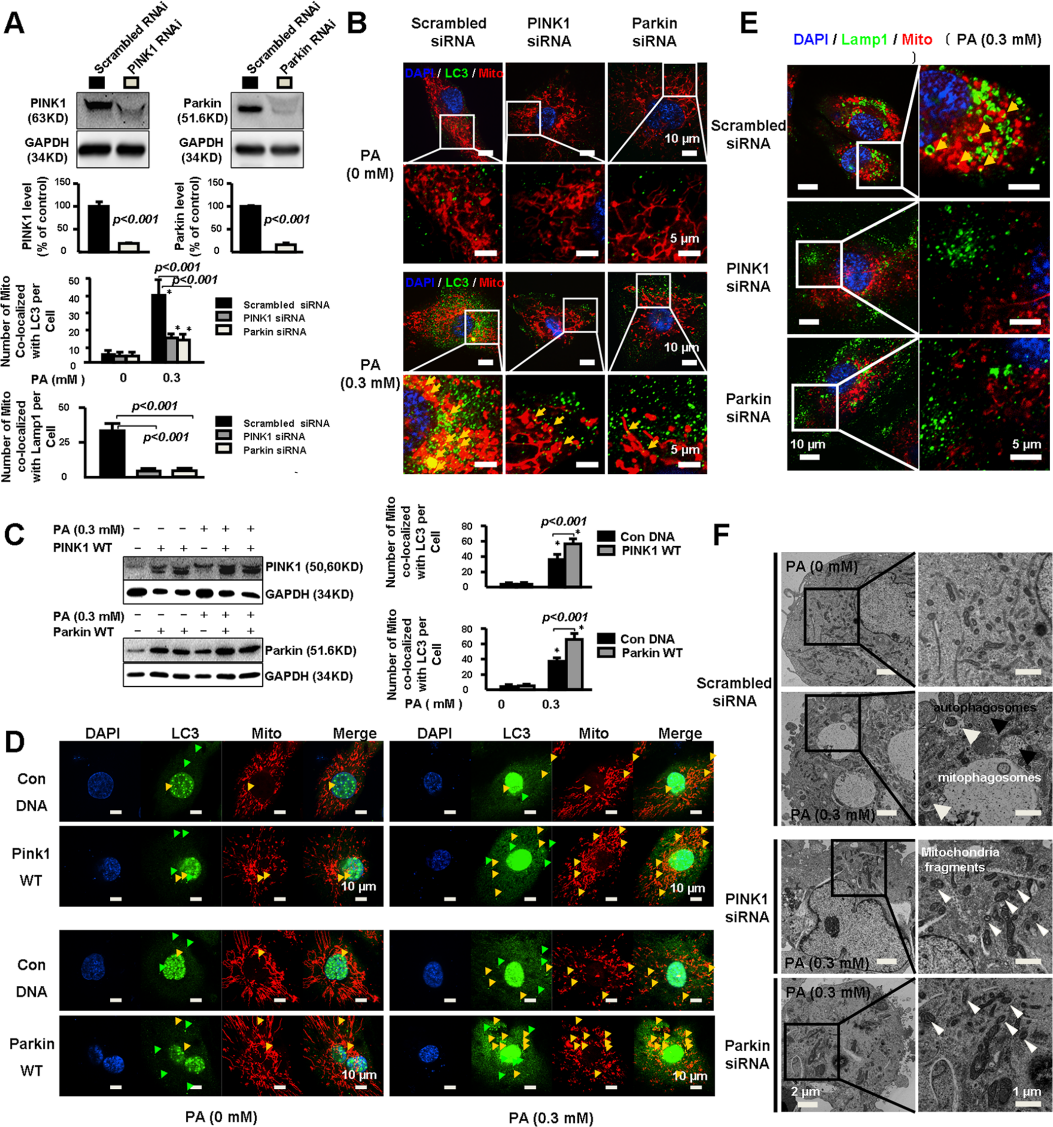
PINK1 and Parkin were involved in mitophagy in PA-treated cells. **A.** HAECs were transfected with PINK1 or Parkin siRNA followed by treatment with PA for 24 hours. Representative western blots and quantitative analysis of 3 independent experiments showed successful silencing of PINK1 and Parkin. **B.** Representative immunostaining images and quantitative analysis of 3 independent experiments showed that PINK1 or Parkin siRNA reduced the number of mitochondria co-localized with LC3 per cell. At least 25–30 cells per condition were analyzed. *P< 0.001 vs. PA (0mM). **C.** HAECs were transfected with PINK1 or Parkin or control plasmid followed by treatment with PA for 48 hours. Representative western blot of 3 independent experiments showed successful overexpression of PINK1 and Parkin. **D.** Representative immunostaining and quantitative analysis of 3 independent experiments showed PA induced LC3 expression and the number of mitochondria co-localized with LC3 per cell which was markedly increased by overexpression of PINK1 or Parkin. At least 25–30 cells per experiment were analyzed. *P< 0.001 vs. PA (0mM). **E.** Representitive immunostaining and quantitative analysis of 3 independent experiments show that silencing PINK1 or Parkin partially prevented PA induced mitochondria co-localized with Lamp1. At least 25–30 cells per experiment were analyzed. **F.** Representative electron microscopy photomicrographs show decreased mitophagy with increased mitochondria fragments in PINK1 or Parkin deficient cells. Black arrows indicated autophagosomes, white arrows indicated mitophagosomes and arrowheads indicated mitochondria fragments.

We then determined the role of PINK1-Parkin in mitophagy induction. Silencing PINK1 or Parkin (Fig 2A) reduced PA-induced increase in LC3 level (Fig 2B), suggesting the involvement of PINK1 and Parkin in autophagy induction. Additionally, PINK1 and Parkin siRNAs reduced PA-induced co-localization of mitochondria with LC3 (Fig 2B), while overexpression of PINK1 or Parkin (Fig 2C) promoted mitochondria-LC3 co-localization (Fig 2D), suggesting the involvement of this pathway in mitophagosome formation. Consistently, silencing PINK1 or Parkin markedly reduced PA induced co-localization of mitochondria with Lamp1, (Fig 2E), suggesting the involvement of this pathway in mitochondrial autolysosome formation. Furthermore, electronic microscopy results showed that knocking down PINK1 or Parkin in PA-treated cells reduced mitophagosomes with accumulation of intracellular mitochondrial fragments (Fig 2F). Together these findings suggest that PINK1 and Parkin are critically involved in mitophagy induction in response to PA challenges.

### PINK1-Parkin protected mitochondrial integrity in PA-treated cells

**Fig 3:**
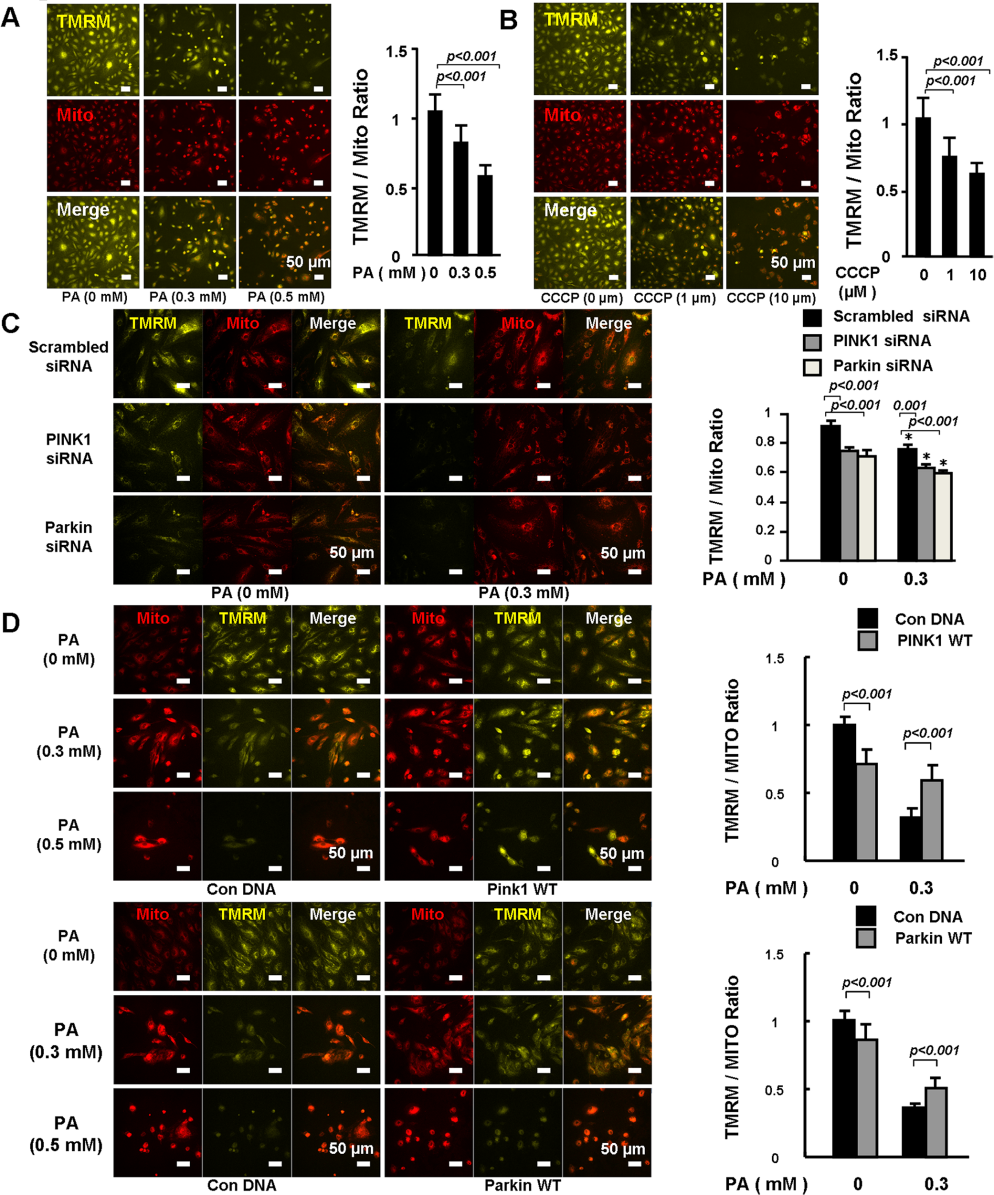
PINK1-Parkin protected mitochondrial integrity. HEACs were treated with PA **(A)** for 24 hours or CCCP (positive control) **(B)** for 6 hours. Active and total mitochondria were stained with TMRM and MitoTracker, respectively. Fluorescent intensity from 20 randomly selected 20 x microscopic fields per group was captured and analyzed. TMRM and Mito ratio was compared. Representative images and quantitative analysis of 3 independent experiments showed that CCCP and PA reduced mitochondrial membrane potential. **C.** HAECs were transfected with PINK1 or Parkin siRNA followed by treatment with PA (0 and 0.3mM) for 24 hours. Fluorescent intensity from 20 randomly selected 20 x microscopic fields per group was captured and analyzed. TMRM and MitoTracker ratio was compared. Representative images and quantitative analysis of 3 independent experiments showed that PINK1 or Parkin siRNA amplified PA-induced reduction in mitochondrial membrane potential. *P< 0.001 vs. PA (0mM). **D.** HAECs were transfected with PINK1 or Parkin or control plasmid followed by treatment with PA (0, 0.3 and 0.5mM) for 48 hours. Fluorescent intensity from 20 randomly selected 20 x microscopic fields per group was captured and analyzed. TMRM and Mito ratio was compared. Representative images and quantitative analysis of 3 independent experiments showed that PINK1 or Parkin overexpression protected PA-induced reduction in mitochondrial membrane potential.

We next examined the protective effect of PINK1 and Parkin on mitochondrial integrity and membrane potential. Tetramethylrhodamine methyl ester (TMRM) and MitoTracker were used to stain the active and total mitochondria, respectively. TMRM/Mito ratio was used as an index of mitochondrial membrane potential. We observed that PA (Fig 3A) and carbonylcyanide-3-chlorophenylhydrazone (CCCP), (Fig 3B) significantly reduced TMRM/Mito ratio, indicating a reduction in mitochondrial membrane potential and mitochondrial damage. Silencing PINK1 or Parkin not only decreased the mitochondrial membrane potential in PA untreated cells (Fig 3C, Left three rows), but also amplified PA induced reduction in mitochondrial membrane potential (Fig 3C, Right three rows). Overexpression of PINK1 or Parkin partially prevented PA-induced reduction of mitochondrial membrane potential, although they decreased membrane potential in unstressed cells (Fig 3D).

### PINK1-Parkin prevented PA-induced mitochondrial dysfunction

**Fig 4:**
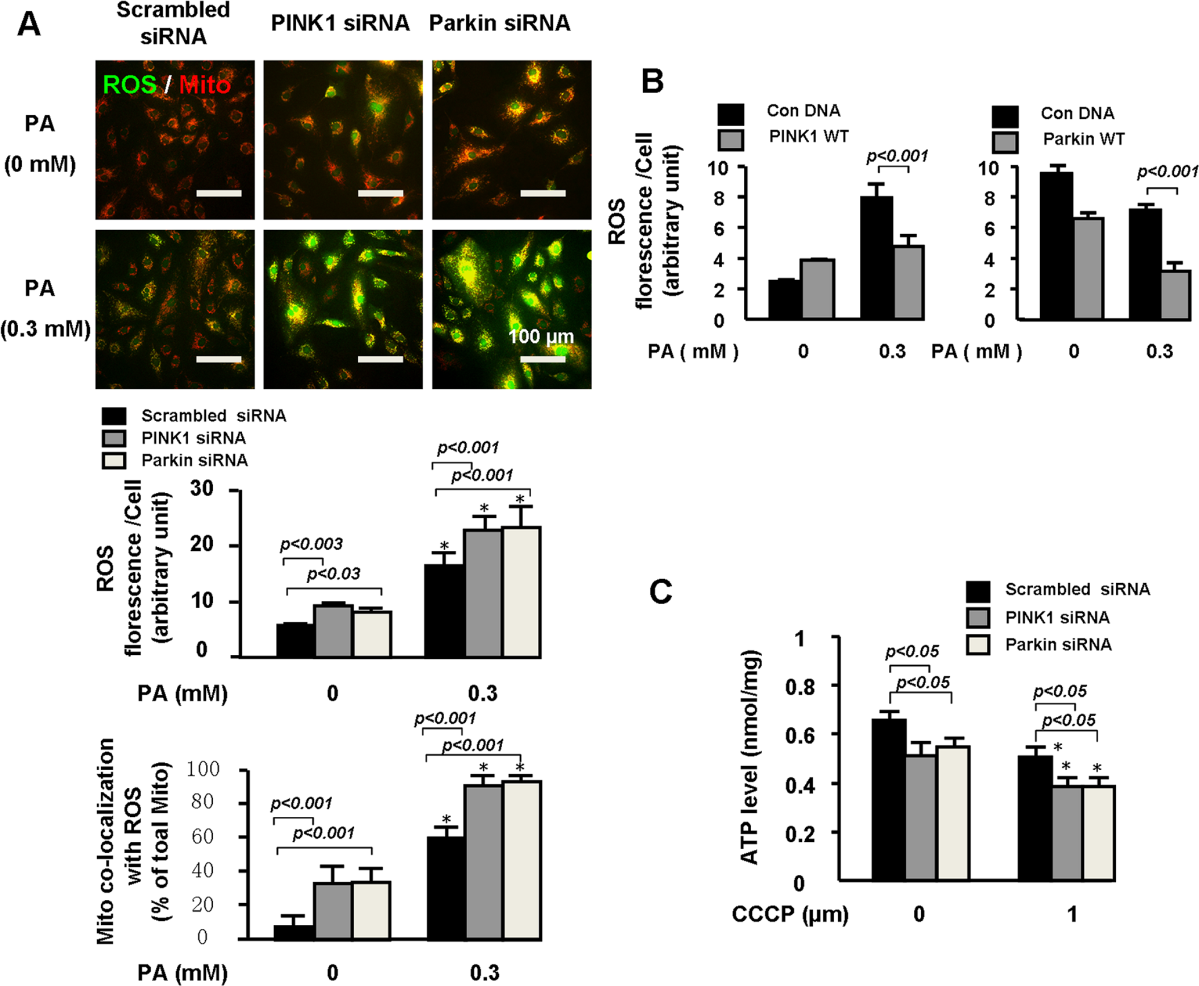
PINK1-Parkin prevented PA-induced ROS accumulation and ATP reduction. HAECs were transfected with PINK1 or Parkin siRNA followed by treatment with PA for 24 hours. **A.** The treated cells were incubated with 50 nM MitoTracker (red) and 10 μM DCFH-DA (green) diluted in serum free culture medium at 37°C for 30 min in the darkness. Intracellular ROS and mitoROS accumulation was compared between the groups. Representative images and quantitative analysis of 3 independent experiments indicated that PA induced intracellular ROS and mitoROS accumulation was increased by PINK1 or Parkin siRNA. **B** HAECs were transfected with PINK1 or Parkin or control plasmid followed by treatment with PA for 48 hours. The treated cells were incubated with 10 μM DCFH-DA (green) diluted in serum free culture medium at 37°C for 30 min in the darkness. Intracellular ROS accumulation was compared between the groups. Representative images and quantitative analysis of 3 independent experiments indicated that PA induced intracellular ROS accumulation was decreased by PINK1 or Parkin overexpression. **C.** ATP levels were measured. Quantitative analysis of 3 independent experiments indicated PINK1 or Parkin siRNA aggravated CCCP-induced ATP reduction.

We then examined the effects of PINK1 and Parkin on PA-induced accumulation of intracellular ROS and mitochondrial ROS (mitoROS). Silencing PINK1 or Parkin increased intracellular ROS and mitoROS levels in unstressed cells (Fig 4A). PA significantly increased intracellular ROS and mitoROS levels, which were increased even more when PINK1 or Parkin was knocked down (Fig 4A). Although overexpressing PINK1 slightly increased intracellular ROS level in cells without PA treatment, PINK1 and Parkin overexpression significantly reduced PA-induced intracellular ROS production (Fig 4B).

We also monitored ATP level to indicate mitochondrial function. However, as an important energy resource, PA at 0.3mM concentration increased ATP level even though it induced mitochondrial damage (data not shown). We therefore used CCCP, an uncoupling protein, to mitochondrial dysfunction and ATP reduction. Treating cells with CCCP for 6 h reduced ATP levels. Silencing PINK1 or Parkin not only decreased ATP level in unstressed cells (Fig 4C), but also magnified ATP reduction in CCCP treated cells, indicating an important role of PINK1 and Parkin in protecting mitochondrial function.

### PINK1-Parkin prevented PA-induced endothelial death

**Fig 5:**
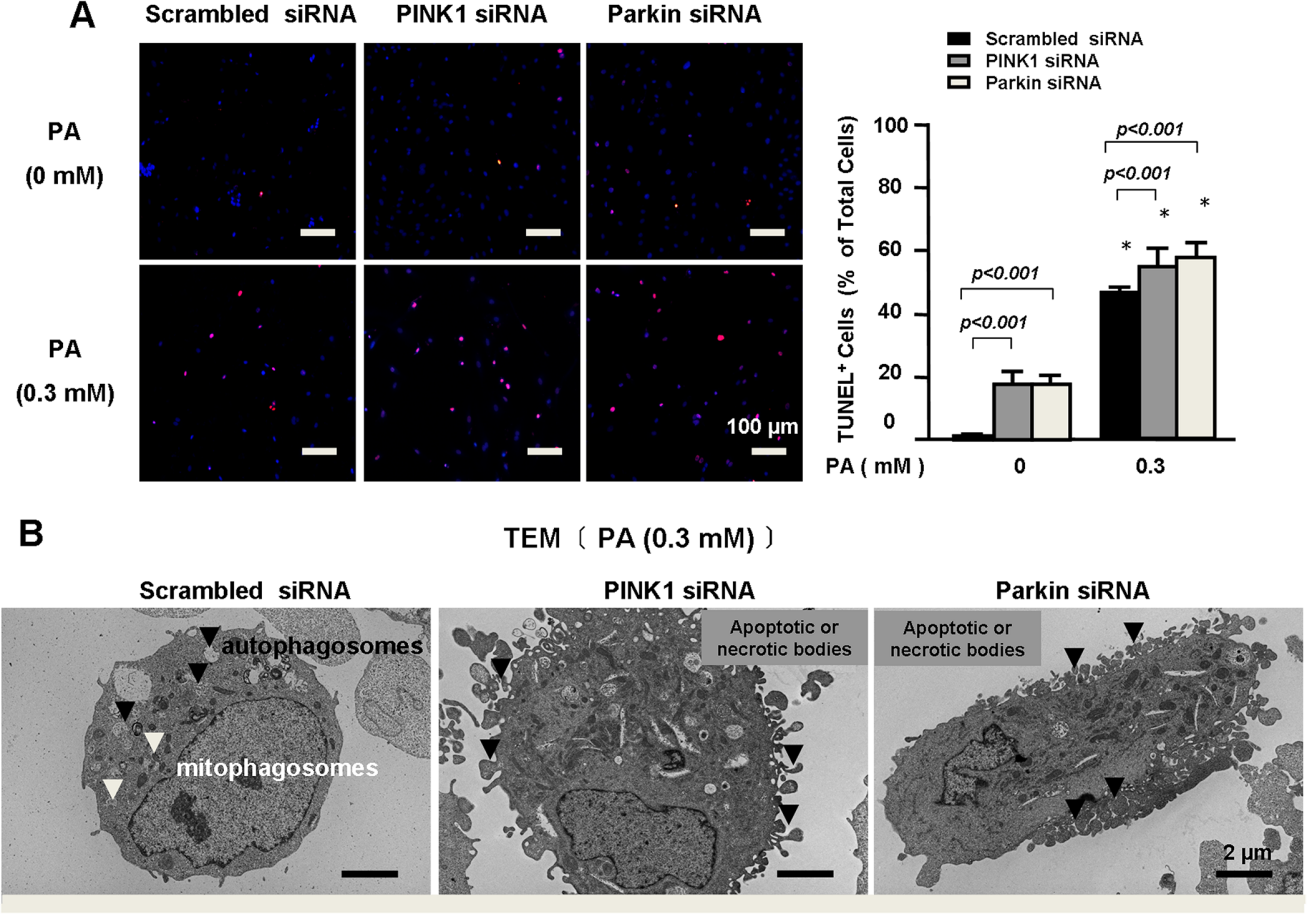
PINK1-Parkin prevented PA-induced endothelial death. **A.** HAECs were transfected with PINK1 or Parkin siRNA followed by treatment with PA for 24 hours. Representative images and quantification of 3 independent TUNEL analysis showed that PINK1 or Parkin siRNA increased PA-induced apoptotic cell death. **B.** Representative electron microscopy photomicrographs of PA-treated cells show that PINK1 or Parkin siRNA increased apoptotic or necrotic bodies.

We further examined the effects of PINK1 and Parkin on PA-induced endothelial damage. We observed that PINK1 or Parkin deficiency induced apoptotic cell death (Fig 5A), which was even more significant in PA treated cells. Electronic microscopy analysis of the PA treated cells also showed that PINK1 or Parkin deficiency increased apoptotic or necrotic bodies (Fig 5B). All these findings supported a critical role of PINK1 and Parkin in preventing PA-induced endothelial injury.

### PINK1-Parkin pathway was activated in blood vessels in obese mice and diabetic mice

**Fig 6:**
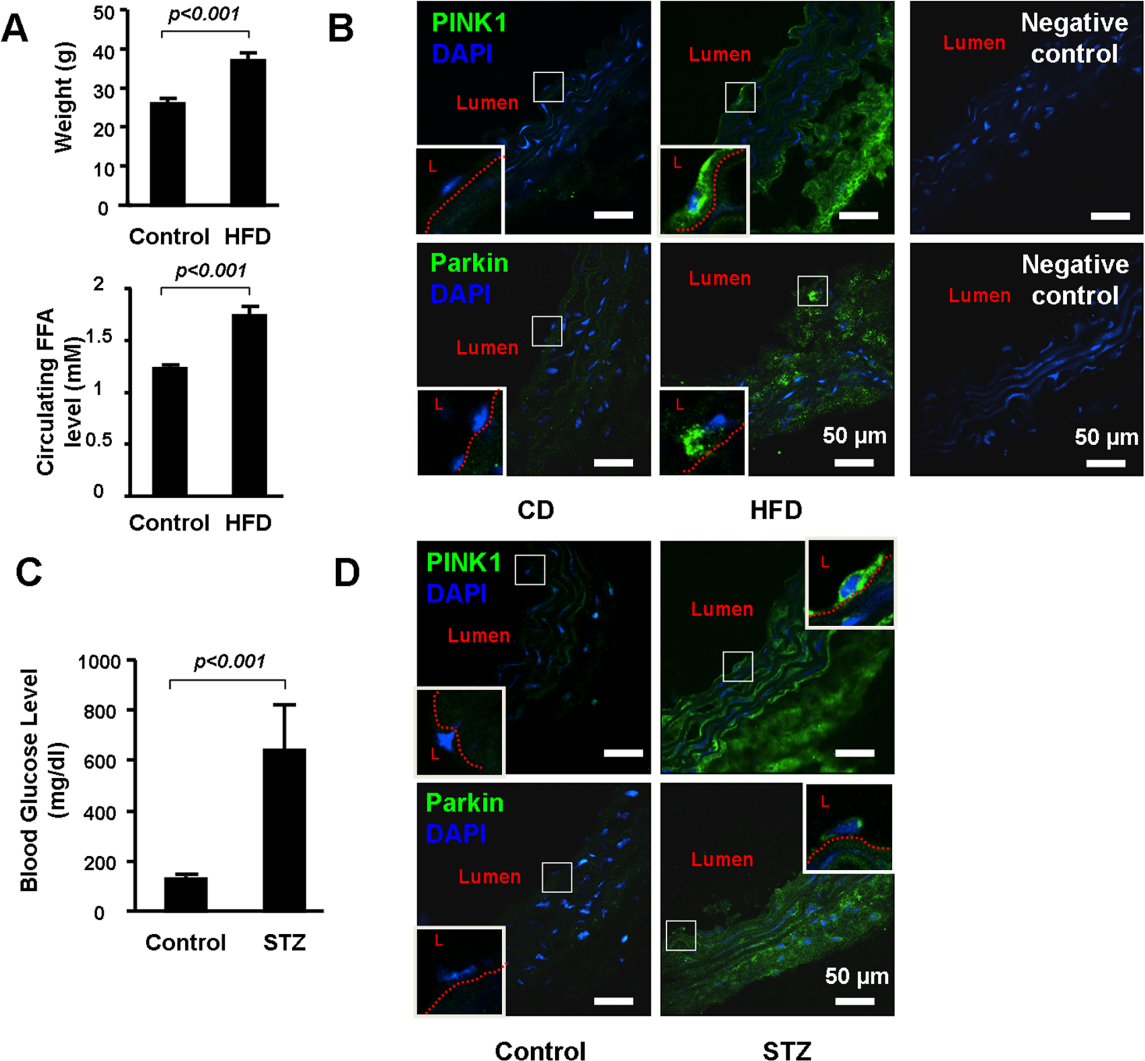
PINK1 and Parkin were up-regulated in the vascular wall of the obese and diabetic mice. **(A)**. Four week old wild-type male WT mice were either fed with chow diet (CD, n = 6) or high fat diet (HFD, n = 12) for 12 weeks. Body weight levels and free fatty acid (FFA) were compared between the groups. **(B)**. Representative immunostaining showed increased PINK1 and Parkin in aortic wall and in endothelial cells (enlarged in the square box) in HFD fed mice. L indicated lumen. Red dot line indicated internal elastic plate of aorta. Negative controls for the PINK1 and Parkin immunostaining were shown on the right. **(C)**. Eight week old wild-type male mice received daily IP injection of 50 mg/kg body weight streptozotocin (STZ, n = 8) or vehicle (n = 5) for five consecutive days. Aortas were collected 4 weeks after the STZ injection. Blood glucose level was compared between the groups. **(D)** Representative immunostaining showed increased PINK1 and Parkin in the aortic wall and in endothelial cells (enlarged in the square box) in STZ treated mice. L indicated lumen. Red dot line indicated internal elastic plate of aorta. Negative controls for the PINK1 and Parkin immunostaining were the same of **(B)**.

Finally, we examined the expression of PINK1-Parkin pathway in blood vessels from high fat diet (HFD)-induced obese mice and in streptozotocin (STZ)-induced diabetic mice. As expected, HFD increased body weight and elevated circulating free fatty acid (FFA) levels (Fig 6A), and STZ treatment caused sustained hyperglycemia (Fig 6C). Immunostaining showed increased PINK1 and Parkin in all layers of the aortic wall including endothelial cells in HFD fed mice (Fig 6B) and STZ treated mice (Fig 6D), indicating the upregulation of PINK1-Parkin pathway in vascular wall of the obese mice and diabetic mice.

## Discussion

In this study, we examined the role of PINK1 and Parkin in protecting mitochondrial structure and functions under metabolic stress. We found that PINK1 and Parkin were upregulated in the vascular wall of the obese mice and diabetic mice, and were activated by palmitic acid treatment in cultured endothelial cells. PINK1 and Parkin, by promoting mitophagy, protected mitochondrial integrity, prevented ROS production and subsequent apoptosis.

Mitochondria are fundamental regulators of endothelial health and function. Different from cells with higher energy demands, ATP supplies in endothelial cells are relatively independent of mitochondrial oxidative pathways. However, mitochondria in endothelial cells play a critical role in generating ROS, modulating intracellular signaling and controlling cell death [10,25]. In metabolic syndrome, mitochondrial dysfunction and ROS overproduction are often observed in endothelial cells [25]. Metabolic stress-induced mitochondrial injury has been shown to contribute to endothelial dysfunction and cardiovascular abnormalities [9,26]. Consistent with previous reports, we observed that PA induced mitochondrial dysfunction and ROS production, which were associated with increased apoptosis in endothelial cells. Preserving mitochondrial function is therefore critically important.

Mitophagy is an important protective mechanism for eliminating damaged mitochondria and preserving healthy mitochondrial population [14]. PINK1—Parkin is a critical pathway in controlling mitophagy. Loss-of-function mutations in PINK1 or Parkin cause mitochondrial dysfunction, and are directly linked to Parkinson’s disease [18,19]. In this study, we show that the PINK1-Parkin pathway was upregulated in obese mice and diabetic mice. In cultured endothelial cells, PA induced significant mitophagy, which was associated with activation of PINK1- Parkin. Knocking down PINK1 or Parkin resulted in impaired mitophagy with intracellular accumulation of damaged mitochondria, and subsequent mitochondrial dysfunction, ROS production and apoptosis. Thus PINK1-Parkin pathway, by promoting mitophagy, plays a critical role in maintaining mitochondrial integrity and protecting endothelial cells under metabolic stress condition. Several other mechanisms such as mitochondrial biogenesis and mitochondrial unfold protein response may also exist to maintain healthy mitochondrial structure. It will be interesting to investigate whether PINK1 and Parkin are also involved in the regulation of these mechanisms.

Interestingly, PA showed dual effects on this pathway in endothelial cells. While PA stimulated PINK1 and Parkin at lower dose, it inhibited this pathway at higher dose. This finding suggests that under certain degree of stress, PINK1- Parkin pathway may be activated in response to mitochondrial injury to remove the damaged mitochondria. However, when the stress is too severe, this protective mechanism may be shut down, leading to accumulation of damaged mitochondria and subsequent cell death. Further studies are required to fully understand how this pathway is regulated under low and high metabolic stress, and whether mitochondrial function can be protected by manipulating the upstream signaling that control the activation of this pathway.

## Conclusions

PINK1 and Parkin pathway are elevated in obese mice and diabetic mice. This pathway appears to be essential for mitochondrial integrity under metabolic stress. PINK1—Parkin mediated mitophagy protects endothelial cells from metabolic stress-induced mitochondrial damage and cell death, hence may provide a novel target for the development of therapeutic strategies in metabolic syndrome.
